# Use of Digital Health Interventions Among Forcibly Displaced People: A Systematic Review and Meta-Analysis

**DOI:** 10.1001/jamanetworkopen.2025.42379

**Published:** 2025-11-12

**Authors:** Sargun Kaur Virk, Rachel Ann Poovathoor, Sanjana Ravi, Nihan Ercanli, Imelda Vetter, Andrew Robert Milewski, Gunisha Kaur

**Affiliations:** 1 Department of Anesthesiology, Weill Cornell Medicine, New York, New York; 2 Brooks School of Public Policy, Cornell University, Ithaca, New York; 3 Department of Pediatrics, Duke University, Durham, North Carolina; 4 Department of Medical Education, University of Texas at Austin

## Abstract

**Importance:**

Forcibly displaced people face significant biopsychosocial barriers to accessing health care. Digital health interventions have emerged as tools to bridge care gaps and have been successfully implemented in other disadvantaged populations to improve health care access and outcomes. However, the feasibility and practicality of digital health interventions among forcibly displaced individuals cannot be fully evaluated without characterizing recruitment and retention rates for these interventions in this unique population.

**Objective:**

To evaluate the recruitment rate and retention rate for digital health interventions in forcibly displaced people.

**Data Sources:**

A comprehensive search was conducted in PubMed, Web of Science, and SocINDEX in January 2023, with an updated search in February 2024. The search strategy followed Preferred Reporting Items for Systematic Reviews and Meta-Analyses guidelines. The protocol was registered in PROSPERO.

**Study Selection:**

Only randomized clinical trials evaluating digital health interventions in forcibly displaced populations were included. Non–peer reviewed studies, reviews, and studies lacking recruitment or retention data were excluded. Two independent reviewers screened and selected studies, and conflicts were resolved by a third reviewer.

**Data Extraction and Synthesis:**

Data were extracted independently by 2 reviewers using Covidence, and a third reviewer resolved discrepancies. Meta-analysis was conducted using a random-effects model to estimate the pooled recruitment rate and retention rate. Study quality was assessed using the Downs and Black Checklist.

**Main Outcomes and Measures:**

Recruitment rate (percentage of prospective participants who were successfully randomized) and retention rate (percentage of randomized participants who completed the intervention).

**Results:**

A total of 9 randomized clinical trials with 2858 participants were analyzed. The most frequently investigated technology was mobile applications (4 trials), followed by mobile phones (3 trials), with 1 study utilizing tablets and another using wearables. Recruitment rates varied widely across studies (16% to 100%), as did retention rates (28% to 91%). The pooled recruitment rate was 91% (95% CI, 57%-99%; *I^2^* = 99%), and the pooled retention rate was 68% (95% CI, 48%-84%; *I^2^* = 98%).

**Conclusions and Relevance:**

In this systematic review and meta-analysis of 9 randomized clinical trials, forcibly displaced populations exhibited variable recruitment and retention rates, although pooled estimates were high. These findings suggest that digital technologies provide a unique opportunity for scalable health interventions in this population, which may mitigate the negative health consequences arising from the many biopsychosocial barriers to in-person care.

## Key Points

**Question:**

What are the recruitment and retention rates for digital health interventions among forcibly displaced people?

**Findings:**

In this systematic review and meta-analysis of 9 randomized clinical trials with 2858 participants, recruitment and retention rates were highly variable, ranging from 16% to 100% and 28% to 91%, respectively. The pooled estimates were high at 91% for recruitment and 68% for retention.

**Meaning:**

These findings suggest that digital technologies—thanks to their low cost, accessibility, and scalability—may provide a feasible way to deliver health interventions in forcibly displaced populations.

## Teaser

This systematic review and meta-analysis examines the recruitment and retention rates of forcibly displaced people in randomized clinical trials using digital health interventions.

## Introduction

As of 2024, an estimated 120 million people worldwide have been forcibly displaced.^1^ Owing to numerous barriers, accessing health care remains a persistent challenge for forcibly displaced people.^2^ However, digital health interventions have been used effectively in other underserved populations^3,4^ and present a unique opportunity to bridge health care gaps. Smartphone ownership among forcibly displaced individuals exceeds 85%,^5^ and the digital literacy rate is high in this population. In a study of 24 asylum applicants, only 1 participant reported not using the internet due to insufficient literacy.^6^

Digital technologies have been leveraged previously to improve various aspects of displaced persons’ lives, including employment, education, and communication.^7,8,9^ Several studies—including work performed by the United Nations—have demonstrated that digital tools can effectively promote health care engagement, enhance health care delivery, and positively alter health behaviors and outcomes in this vulnerable population.^3,10,11^ However, little is known about the ability to recruit and retain forcibly displaced people in digital health interventions, factors that are critical for determining the feasibility and practicality of these interventions broadly and globally. In this meta-analysis, we identify and analyze the recruitment and retention rates of digital health intervention studies in forcibly displaced people.

## Methods

This systematic review adheres to the Preferred Reporting Items for Systematic Reviews and Meta-Analyses (PRISMA) reporting guidelines and is registered in the International Prospective Register of Systematic Reviews (PROSPERO) (CRD42022377195). After developing and validating the search strategy (eTable 1 in Supplement 1), a health sciences librarian (I.V.) conducted searches across PubMed (NLM), Web of Science (Clarivate), and SocINDEX (EBSCOhost) in January 2023, with an update performed in February 2024 to identify articles that were published in the intervening time. The reference lists for the studies that met the inclusion criteria were also screened for relevant citations.

Included studies were randomized clinical trials that evaluated a digital health intervention in groups that met the United Nations High Commissioner for Refugees (UNHCR) definition for forcibly displaced populations.^12^ Only peer-reviewed studies in English were included, and conference abstracts, dissertations, reviews, book chapters, and gray literature were excluded. Digital health tools were defined as technologies—including mobile applications, smartphones, text messaging or short message service texts, tablets, wearable devices, and telehealth platforms—that were used in health care delivery or research. There were no restrictions on the year of publication.

### Statistical Analysis

Abstracts and full texts were screened for inclusion in Covidence—an online software for systematic reviews—by 2 independent reviewers (R.P., S.R., and N.E.). A third reviewer (S.V. and R.P.) resolved any conflicts. The study design, population characteristics, number of participants (prospective, randomized, and providing the outcome of interest), intervention details, and investigated outcomes were extracted from each study. In the single study that reported data for households, each household was counted as an individual participant to maintain consistency in the pooled analysis.^13^ Because the intervention for 1 study comprised a 1-time interaction with participants, this study was excluded from the meta-analysis of retention rates.^14^ A random-effects model using the inverse-variance method was employed to estimate pooled recruitment and retention rates, and a logit transformation was used to stabilize variances. All calculations were performed in R version 4.1.1 (R Project for Statistical Computing). To assess each study’s influence on the pooled estimates, a leave-one-out sensitivity analysis was conducted wherein each study was sequentially excluded from the meta-analysis. The risk for bias was assessed for each study using the Downs and Black Checklist,^15^ and scores were categorized as follows: excellent (26 or above), good (20 to 25), fair (15 to 19), and poor (14 or below).^16^

## Results

The search strategy identified 617 unique articles from 3 databases, and 9 articles met the predetermined inclusion criteria (eFigure 1 in Supplement 1).^13,14,17,18,19,20,21,22,23^ In total, 2858 unique participants from 8 countries were enrolled across all studies, and the mean age of all the participants was 29.8 years (Table). The most frequently studied primary outcome was mental health (4 studies),^14,17,18,23^ followed by vaccination (2 studies).^13,20^ Among the 8 studies for which retention rates could be evaluated, the length of the interventions ranged from 4 to 52 weeks. Mobile application was the most frequently used technology (4 studies),^17,18,20,23^ followed by providing mobile phones with only basic functionality (3 studies),^13,19,22^ and 1 study each utilized tablets^14^ or wearables.^21^

**Table.** Detailed Characteristics of Included Studies

Source	Study design	Host country	Mean age, y	Intervention	Length of intervention, wk	Recruitment rate, %	Retention rate, %	Outcome
Ahmad et al,^14^ 2012	Pilot RCT	Canada	37.6	Tablet; multi-risk CaPRA tool	0	78	NA	Intention to visit a psychosocial counselor
Böge et al,^17^ 2022	RCT	Germany	28.6	Mobile app; SCCM	12	88	62	Depressive symptom score
Cujipers et al,^18^ 2022	RCT	Lebanon	31.5	Mobile app; Step-by-Step enhanced care as usual	8	75	47	Depressive symptom score, functional impairment
Evans et al,^19^ 2021	Feasibility RCT	US	37.18	Mobile	32	100	71	Possession of health insurance
Frick et al,^20^ 2023	Feasibility and efficacy RCT	Germany	24.3	Mobile app; with short video clips to explain biological basis of COVID-19	6	16	28	Knowledge of COVID-19 and vaccine readiness
Grijalva-Eternod et al,^13^ 2023	RCT	Somalia (IDP)	31.7	Mobile; conditional cash transfer and audio messages	4	54	91	Vaccination
Kim et al,^21^ 2023	Pilot RCT	Republic of Korea	43.2	Wearable activity tracking device	12	100	68	Step count, other metabolic parameters
Logie et al,^22^ 2023	RCT	Uganda	20	Mobile SMS bidirectional messaging	52	100	71	Self-reported HIV uptake and correct status knowledge
Röhr et al,^23^ 2021	RCT	Germany	33	Mobile app; Sanadak app	17	96	87	PTSD

Abbreviations: CaPRA, computer-assisted Psychosocial Risk Assessment; IDP, internally displaced people; NA, not applicable; PTSD, posttraumatic stress disorder; RCT, randomized clinical trial; SCCM, Stepped Care and Collaborative Model; SMS, short message service.

Most studies incorporated modules for information dissemination, primarily through audio and video clips,^13,17,18,20,22^ and 1 study provided individualized counseling.^21^ One study provided a smartphone with broadband service and preloaded applications to improve connectivity, but did not include any structured instructional or health modules.^19^ To enhance recruitment, all studies incorporated community engagement strategies, including collaborations with local organizations, outreach efforts, and culturally tailoring the messaging to ensure that the digital health interventions resonated with the target population.

To encourage retention, various push factors were employed to sustain engagement, including automated notifications, incentive-based rewards, and digital support mechanisms such as e-helpers or mobile mentors who provided technological assistance. Two studies integrated gamification features to enhance interactivity.^20,23^ All interventions were culturally and linguistically adapted to ensure that materials were accessible and aligned with the participants’ needs. Notably, the study with the highest retention rate delivered a 2-minute narrative on health and nutrition twice weekly, followed by a 1-minute message to reinforce participant engagement.^13^

**Figure 1.**
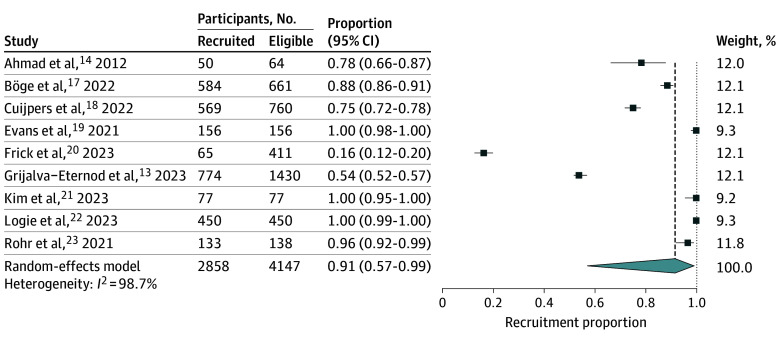
Pooled Recruitment Rate

**Figure 2.**
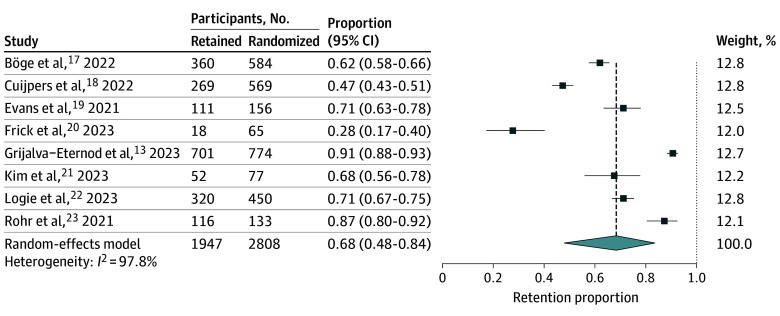
Pooled Retention Rate

Recruitment rates varied widely, from 16% to 100% across studies, and retention rates ranged from 28% to 91%. The pooled recruitment rate was 91% (95% CI, 57%-99%; *I^2^* = 99%) and the pooled retention rate was 68% (95% CI, 48%-84%; *I^2^* = 98%) (Figures 1 and 2). Leave-one-out sensitivity analysis demonstrated stable pooled estimates (recruitment rate, 86%-94%; retention rate, 63%-73%) (eFigures 2 and 3 in Supplement 1).

For recruitment rate, because 3 studies reported 100% recruitment, we conducted an additional sensitivity analysis excluding these 3 studies.^19,21,22^ The pooled recruitment rate was decreased to 73% (95% CI, 32%-94%; *I^2^* = 99%), with leave-one-out estimates ranging from 64% to 82%, confirming that recruitment rate remained moderate to high despite substantial heterogeneity (eFigures 4 and 5 in Supplement 1). According to the Downs and Black Checklist, the quality of 5 studies was rated as good^13,17,18,21,23^ and the quality of 4 studies was rated as fair^14,19,20,22^ (eTable 2 in Supplement 1).

## Discussion

Recruitment and retention rates for digital health interventions among forcibly displaced people were highly variable, ranging from 16% to 100% and 28% to 91%, respectively. The pooled estimates showed high recruitment and retention rates, 91% and 68%, respectively, although the substantial heterogeneity may reflect differences in study design, intervention type, and population context. Notably, sensitivity analysis excluding the 3 studies that reported 100% recruitment yielded a lower pooled estimate of 73%, yet recruitment remained moderate to high. Together, these findings highlight both the potential for digital tools to provide low-cost, accessible, and scalable mechanisms to improve health care access in this traditionally hard-to-reach population, and the importance of accounting for contextual variability in their implementation.

Our pooled estimates contrast with the low recruitment and retention rates that are consistently reported for digital health trials in general populations.^24^ A review of 37 remote digital health studies conducted across multiple countries and disease areas found that the median completion rate was approximately 50% in general populations, which was considered relatively high given that retention rates as low as 10% are common in digital health research.^25^ Similarly, a cross-study evaluation of more than 100 000 US participants enrolled in 8 app-based digital health studies across multiple disease areas found retention rates ranging from 39% to 68%.^26^

The comparatively high recruitment and retention rates in our study may be attributable to greater digital literacy, particularly among younger individuals. Indeed, the mean age of the participants in our study was 29.8 years, aligning with the demographic profile of the global refugee population, wherein younger individuals constitute the majority.^27^ These findings challenge the assumption that digital engagement may be inherently low in vulnerable populations. Rather, retention in digital interventions is likely highly context-dependent and influenced by population and sociodemographic factors.^28^

### Limitations

This review has several limitations. First, the search was restricted to only English-language studies, introducing potential selection bias. Second, the limited number of studies precluded the assessment of publication bias. Lastly, meta-regression was not feasible due to the small number of available studies.

## Conclusions

In this systematic review and meta-analysis, recruitment and retention rates for digital health interventions in forcibly displaced populations were variable, but pooled estimates were high, underscoring an opportunity for digital tools to reduce the health impact of the many biopsychosocial barriers to in-person care. However, digital tools should ideally complement in-person engagement, which is instrumental for effective health promotion. Future studies should evaluate the effectiveness of digital technologies in improving health outcomes and should optimize intervention strategies for maximum, sustained impact, while also addressing barriers to in-person engagement.

## Notes

Supplement 1. eTable 1. PubMed Search Strategy eFigure 1. PRISMA Flowchart eFigure 2. Leave-One-Out Analysis for Pooled Recruitment Rate eFigure 3. Leave-One-Out Analysis for Pooled Retention Rate eFigure 4. Pooled Recruitment Rate Excluding Three Studies With 100% Recruitment Rate eFigure 5. Leave-One-Out Analysis for Pooled Recruitment Rate Excluding Three Studies With 100% Recruitment Rate eTable 2. Downs and Black Checklist

Supplement 2. Data Sharing Statement
