# Supplementary material for: Use of Digital Health Interventions Among Forcibly Displaced People: A Systematic Review and Meta-Analysis
Source: JAMA Netw Open. 2025 Nov 12;8(11):e2542379. doi: 10.1001/jamanetworkopen.2025.42379 (PMC12612957; doi:10.1001/jamanetworkopen.2025.42379)
Supplement: Supplement 1. — eTable 1. PubMed Search Strategy eFigure 1. PRISMA Flowchart eFigure 2. Leave-One-Out Analysis for Pooled Recruitment Rate eFigure 3. Leave-One-Out Analysis for Pooled Retention Rate eFigure 4. Pooled Recruitment Rate Excluding Three Studies With 100% Recruitment Rate eFigure 5. Leave-One-Out Analysis for Pooled Recruitment Rate Excluding Three Studies With 100% Recruitment Rate eTable 2. Downs and Black Checklist [file jamanetwopen-e2542379-s001.pdf]

Supplemental Online Content

Virk SK, Poovathoor RA, Ravi S, et al. Use of digital health interventions among forcibly displaced people. *JAMA Netw Open.* 2025;8(11):e2542379.
doi:10.1001/jamanetworkopen.2025.42379

eTable 1. PubMed Search Strategy

eFigure 1. PRISMA Flowchart

eFigure 2. Leave-One-Out Analysis for Pooled Recruitment Rate

eFigure 3. Leave-One-Out Analysis for Pooled Retention Rate

eFigure 4. Pooled Recruitment Rate Excluding Three Studies With 100% Recruitment Rate

eFigure 5. Leave-One-Out Analysis for Pooled Recruitment Rate Excluding Three Studies With 100% Recruitment Rate

eTable 2. Downs and Black Checklist

This supplemental material has been provided by the authors to give readers additional information about their work.

© 2025 Virk SK et al. *JAMA Network Open*.

eTable1. PubMed Search Strategy

**A - refugee**	Refugee* OR "Asylum seeker*" OR "Displaced person*" OR "Displaced people" OR "Displaced population*" OR "Stateless people" OR "Stateless person*" OR "Internally displaced" OR "Externally displaced" OR "Undocumented immigrant*" OR "Undocumented worker*" OR "Undocumented alien*" OR "Illegal immigrant*" OR "Unauthorized immigrant*" OR “refugees”[MeSH] OR “refugee camps”[MeSH] OR “undocumented immigrants”[MeSH]
**B – digital intervention**	“Digital technolog*"[Title/Abstract] OR "Digital health"[Title/Abstract] OR mHealth[Title/Abstract] OR m-health[Title/Abstract] OR "mobile health"[Title/Abstract] OR SMS[Title/Abstract] OR "short message service"[Title/Abstract] OR "Text messag*"[Title/Abstract] OR Texting[Title/Abstract] OR "cell phone*"[Title/Abstract] OR "Cellular phone*"[Title/Abstract] OR "Mobile phone*"[Title/Abstract] OR "Mobile telephone*"[Title/Abstract] OR Smartphone*[Title/Abstract] OR Smart-phone*[Title/Abstract] OR "Smart phone*"[Title/Abstract] OR Iphone*[Title/Abstract] OR i-phone*[Title/Abstract] OR ehealth[Title/Abstract] OR e-health[Title/Abstract] OR Telemedicine[Title/Abstract] OR Telehealth[Title/Abstract] OR Tele-health[Title/Abstract] OR mobile app[Title/Abstract] OR mobile apps[Title/Abstract] OR mobile application*[Title/Abstract] OR smartphone app[Title/Abstract] OR smartphone apps[Title/Abstract] OR smartphone application*[Title/Abstract] OR "Electronic mail*"[Title/Abstract] OR Email*[Title/Abstract] OR "hand-held device*"[Title/Abstract] OR "handheld device*"[Title/Abstract] OR "palm pilot*"[Title/Abstract] OR "portable device*"[Title/Abstract] OR "mobile device*"[Title/Abstract] OR tablet*[Title/Abstract] OR PDA[Title/Abstract] OR PDAs[Title/Abstract] OR "Personal digital assistant*"[Title/Abstract] OR "Wearable technolog*"[Title/Abstract] OR "Wearable device*"[Title/Abstract] OR "Wearable electronics"[Title/Abstract] OR "Wearable electronic device*"[Title/Abstract] OR “telemedicine”[MeSH] OR “cell phone”[MeSH] OR “computers, handheld”[MeSH] OR “wearable electronic devices”[MeSH]
**Search**	A AND B
**Filters**	English[Language]

© 2025 Virk SK et al. *JAMA Network Open*.

eFigure 1. PRISMA Flowchart

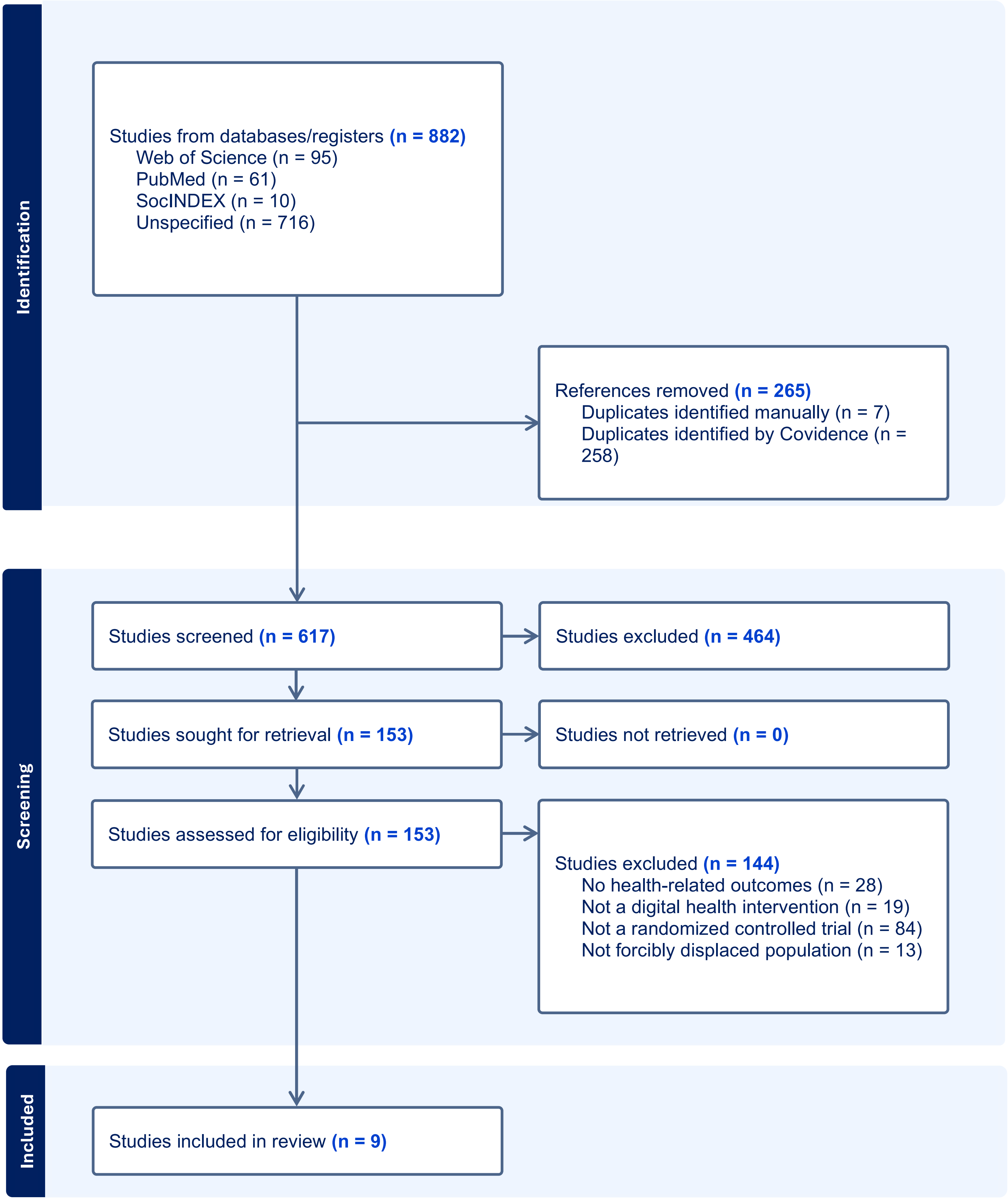

© 2025 Virk SK et al. *JAMA Network Open*.

eFigure 2. Leave-One-Out Analysis for Pooled Recruitment Rate

	proportion	95%-CI	p-value	tau^2	tau	I^2
Omitting Ahmad_2012	0.9273	[0.5323; 0.9931]		7.4568	2.7307	98.8%
Omitting Boge_2022	0.9206	[0.5008; 0.9926]		7.6321	2.7626	98.3%
Omitting Cuijpers_2022	0.9290	[0.5410; 0.9932]		7.3892	2.7183	98.7%
Omitting Evans_2021	0.8803	[0.4568; 0.9847]		5.7702	2.4021	98.8%
Omitting Frick_2023	0.9441	[0.7053; 0.9917]		4.4458	2.1085	97.8%
Omitting Grijalva-Eternod_2023	0.9358	[0.5894; 0.9933]		6.7940	2.6065	98.7%
Omitting Kim_2023	0.8895	[0.4558; 0.9872]		6.3264	2.5152	98.8%
Omitting Logie_2023	0.8644	[0.4686; 0.9788]		4.7656	2.1830	98.8%
Omitting Rohr_2021	0.9068	[0.4623; 0.9910]		7.3685	2.7145	98.7%
Pooled estimate	0.9139	[0.5681; 0.9885]		6.4274	2.5352	98.7%

© 2025 Virk SK et al. *JAMA Network Open*.

eFigure 3. Leave-One-Out Analysis for Pooled Retention Rate

	proportion	95%-CI	p-value	tau^2	tau	I^2
Omitting Boge_2022	0.6926	[0.4487; 0.8618]		1.1647	1.0792	98.1%
Omitting Cuijpers_2022	0.7107	[0.4849; 0.8651]		1.0219	1.0109	97.1%
Omitting Evans_2021	0.6792	[0.4323; 0.8548]		1.1760	1.0844	98.1%
Omitting Frick_2023	0.7315	[0.5600; 0.8536]		0.6550	0.8093	97.9%
Omitting Grijalva-Eternod_2023	0.6346	[0.4336; 0.7976]		0.7224	0.8499	95.3%
Omitting Kim_2023	0.6844	[0.4383; 0.8577]		1.1750	1.0840	98.1%
Omitting Logie_2023	0.6791	[0.4319; 0.8549]		1.1790	1.0858	98.1%
Omitting Rohr_2021	0.6484	[0.4252; 0.8213]		0.9368	0.9679	98.0%
Pooled estimate	0.6834	[0.4784; 0.8356]		1.0021	1.0011	97.8%

© 2025 Virk SK et al. *JAMA Network Open*.

eFigure 4. Pooled Recruitment Rate Excluding Three Studies With 100% Recruitment Rate

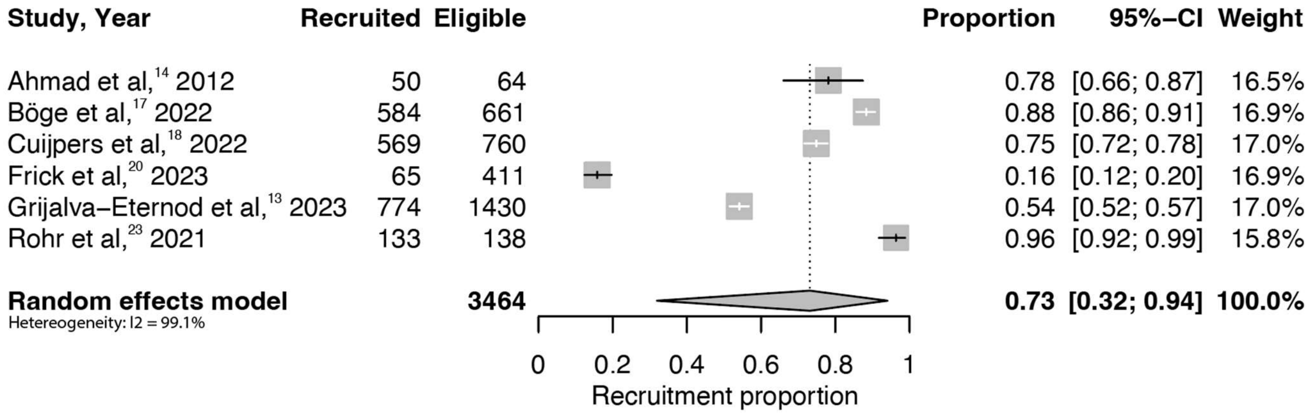

© 2025 Virk SK et al. *JAMA Network Open*.

eFigure 5. Leave-One-Out Analysis for Pooled Recruitment Rate Excluding Three Studies With 100% Recruitment Rate

	proportion	95%-CI	tau^2	tau	I^2
Omitting Ahmad_2012	0.7218	[0.2035; 0.9634]	3.4009	1.8442	99.3%
Omitting Boge_2022	0.6893	[0.1939; 0.9534]	3.0984	1.7602	98.9%
Omitting Cuijpers_2022	0.7289	[0.2073; 0.9651]	3.4392	1.8545	99.2%
Omitting Frick_2023	0.8204	[0.5252; 0.9496]	1.1990	1.0950	98.6%
Omitting Grijalva-Eternod_2023	0.7643	[0.2532; 0.9688]	3.2313	1.7976	99.2%
Omitting Rohr_2021	0.6393	[0.2325; 0.9121]	2.0039	1.4156	99.2%
Random effects model	0.7313	[0.3194; 0.9404]	2.7248	1.6507	99.1%

© 2025 Virk SK et al. *JAMA Network Open*.

eTable2. Downs and Black Checklist

#	Question<br>(Yes = 1; No = 0, unless otherwise stated)	Ahmad 2012	Boge 2022	Cujipers 2022	Rohr 2021	Evans 2021	Grijalva 2023	Frick 2023	Kim 2023	Logie 2023
1	Is the hypothesis/aim/objective of the study clearly described?	1	1	1	1	1	1	1	1	1
2	Are the main outcomes to be measured clearly described in the Introduction or Methods section?	1	1	1	1	1	1	1	1	1
3	Are the characteristics of the patients included in the study clearly described?	1	1	1	1	1	1	1	1	1
4	Are the interventions of interest clearly described?	1	1	1	1	1	1	1	1	
5	Are the distributions of principal confounders in each group of subjects to be compared clearly described?	1	0	0	0	1	1	1	1	1
6	Are the main findings of the study clearly described?	1	1	1	1	1	1	1	1	1
7	Does the study provide estimates of the random variability in the data for the main outcomes?	1	1	1	1	1	1	1	1	1
8	Have all important adverse events that may be a consequence of the intervention been reported?	0	1	1	0	0	1	1	0	
9	Have the characteristics of patients lost to follow-up been described?	0	1	1	0	0	1	1	1	1
10	Have actual probability values been reported (e.g. 0.035 rather than <0.05) for the main outcomes except where the probability value is less than 0.001?	1	1	1	1	1	1	1	1	0
11	Were the subjects asked to participate in the study representative of the entire population from which they were recruited?	0	0	0	1	0	1	1	1	1
12	Were those subjects who were prepared to participate representative of the entire population from which they were recruited?	0	0	0	0	0	1	0	1	1
13	Were the staff, places, and facilities where the patients were treated, representative of the treatment the majority of patients receive?	0	1	1	1	1	1		0	0
14	Was an attempt made to blind study subjects to the intervention they have received?	0	0	1	0	0	0	0	0	0
15	Was an attempt made to blind those measuring the main outcomes of the intervention?	1	1	0	1	0	0	0	0	0
16	If any of the results of the study were based on "data dredging", was this made clear?	1	1	1	1	1	1	0	1	
17	In trials and cohort studies, do the analyses adjust for different lengths of follow-up of patients, or in case-control studies, is the time period between the intervention and outcome the same for cases and controls?	0	1	1	1	0	1	1	1	
18	Were the statistical tests used to assess the main outcomes appropriate?	1	1	1	1	1	1	1	1	1

© 2025 Virk SK et al. *JAMA Network Open*.

#	Question<br>(Yes = 1; No = 0, unless otherwise stated)	Ahmad 2012	Boge 2022	Cujipers 2022	Rohr 2021	Evans 2021	Grijalva 2023	Frick 2023	Kim 2023	Logie 2023
19	Was compliance with the intervention/s reliable?	1	1	1	1	0	0	0	1	0
20	Were the main outcome measures used accurate (valid and reliable)?	1	1	1	1	1	1	1	1	1
21	Were the patients in different intervention groups (trials and cohort studies) or were the cases and controls (case-control studies) recruited from the same population?	1	1	1	1	1	1	1	1	1
22	Were study subjects in different intervention groups (trials and cohort studies) or were the cases and controls (case-control studies) recruited over the same period of time?	0	1	1	1	1	1	1	1	1
23	Were study subjects randomized to intervention groups?	1	1	1	1	1	1	1	1	1
24	Was the randomized intervention assignment concealed from both patients and health care staff until recruitment was complete and irrevocable?	1	0	0	0	0	0	0	0	0
25	Was there adequate adjustment for confounding in the analyses from which the main findings were drawn?	0	0	1	0	0	0	0	1	1
26	Were losses of patients to follow-up taken into account?	0	1	1	1	0	1	1	1	1
27	Did the study have sufficient power to detect a clinically important effect where the probability value for a difference being due to chance is less than 5%?	0	1	1	1	0	1	1	0	1
	**Total score**	**16**	**21**	**22**	**20**	**15**	**22**	**19**	**21**	**17**

© 2025 Virk SK et al. *JAMA Network Open*.
