# Supplementary material for: Use of Digital Health Interventions Among Forcibly Displaced People: A Systematic Review and Meta-Analysis
Source: JAMA Netw Open. 2025 Nov 12;8(11):e2542379. doi: 10.1001/jamanetworkopen.2025.42379 (PMC12612957; doi:10.1001/jamanetworkopen.2025.42379)
Supplement: Supplement 2. — Data Sharing Statement [file jamanetwopen-e2542379-s002.pdf]

Data Sharing Statement

Virk. Use of Digital Health Interventions Among Forcibly Displaced People. *JAMA Netw Open*. Published November 12, 2025. doi:10.1001/jamanetworkopen.2025.42379

Data
Data available: Yes
Data types: Data (not involving human participants), Data dictionary
How to access data: sav4017@med.cornell.edu
When available: With publication

Supporting Documents
Document types: Statistical/analytic code
How to access documents: sav4017@med.cornell.edu
When available: With publication

Additional Information
Who can access the data: anyone requesting the data
Types of analyses: for any purpose
Mechanisms of data availability: with investigator support
